# Attitudes towards assisted suicide and euthanasia among care-dependent older adults (50+) in Austria: the role of socio-demographics, religiosity, physical illness, psychological distress, and social isolation

**DOI:** 10.1186/s12910-017-0233-6

**Published:** 2017-12-07

**Authors:** Erwin Stolz, Hannes Mayerl, Peter Gasser-Steiner, Wolfgang Freidl

**Affiliations:** 1 Institute of Social Medicine and Epidemiology, Medical University of Graz, Universitätsstraße 6/I, 8010 Graz, Austria; 2 Department of Sociology, University of Graz, Universitätsstraße 15/IV, 8010 Graz, Austria

**Keywords:** Care-dependent older adults, Euthanasia, Assisted suicide, Attitudes, Determinants

## Abstract

**Background:**

Care-dependency constitutes an important issue with regard to the approval of end-of-life decisions, yet attitudes towards assisted suicide and euthanasia are understudied among care-dependent older adults. We assessed attitudes towards assisted suicide and euthanasia and tested empirical correlates, including socio-demographics, religiosity, physical illness, psychological distress and social isolation.

**Methods:**

A nationwide cross-sectional survey among older care allowance recipients (50+) in private households in Austria was conducted in 2016. In computer-assisted personal interviews, 493 respondents were asked whether or not they approved of the availability of assisted suicide and euthanasia in case of long-term care dependency and whether or not they would consider using assisted suicide or euthanasia for themselves. Multiple logistic regression analysis was used to assess the impact of potential determinants of attitudes towards assisted suicide and euthanasia.

**Results:**

About a quarter (24.8-26.0%) of the sampled care-dependent older adults approved of the availability of assisted suicide and euthanasia respectively indicated the will to (hypothetically) make use of assisted suicide or euthanasia. Attitudes towards assisted suicide were most favourable among care-dependent older adults living in urban areas, those who did not trust physicians, those who reported active suicide ideation, and individuals with a strong fear of dying. With regard to euthanasia, living alone, religiosity and fear of dying were the central determinants of acceptance.

**Conclusions:**

Positive attitudes towards and will to (hypothetically) use assisted suicide and euthanasia were expressed by a substantial minority of care-dependent older adults in Austria and are driven by current psychological suffering and fear of the process of dying in the (near) future. Community-based psychosocial care should be expanded to address psychological distress and fears about end-of-life issues among care-dependent older adults.

**Electronic supplementary material:**

The online version of this article (10.1186/s12910-017-0233-6) contains supplementary material, which is available to authorized users.

## Background

Against the background of higher life expectancy and higher risk of suffering from chronic diseases [1], medical advancements regarding artificial life extension and not least acts of legalisation in a number of countries [2], end-of-life decisions have emerged as a controversially disputed ethical and legal issue in many European countries. Among end-of-life decisions, euthanasia (EUT) – the voluntary, patient-requested and deliberate ending of life by a physician administering a lethal substance – and assisted suicide (AS) – taking one’s own life but requiring the help of another person, often a physician, to do so – are among the most controversial. In Europe, EUT and physician-AS are currently legal under specific conditions in Belgium, Luxembourg and the Netherlands [2–4]. Additionally, physician-AS, but not EUT, is also legal in Switzerland, and assistance in suicide from relatives and other lay-persons is under certain conditions not subject to prosecution in a few more countries (e.g. Germany, Switzerland, Finland). In most European countries, however, both acts constitute punishable crimes. In Austria, for example, EUT is considered a criminal offence according to §77 StGB (Austrian Criminal Code), punishable with prison sentences ranging from 6 months to 5 years.

Irrespective of their legal status in most European countries, public discourse on both AS and EUT has been extensive and polarising in many European countries in the last years. In such public discussions, which often spark around or are fuelled by the media coverage of individual cases (e.g. [5–8]), survey studies assessing attitudes towards end-of-life decision can serve as empirical anchor points. In this study, we focussed on attitudes towards AS and EUT among care-dependent older adults – that is, older adults who require care and assistance from others with activities of daily living – and how these attitudes can be explained.

### Attitudes towards end-of-life decisions in the context of long-term care dependency

In survey studies – as in public discourse – discussed scenarios of end-of-life decisions often involve terminal conditions such as late-stage cancer, severe dementia or uncontrollable pain (e.g. [9–13]). Fewer studies confront survey respondents with vignettes including scenarios of need for long-term care or the feeling of being a burden to others for which respondents should express their approval or rejection. In such studies, however, a considerable minority of the population emphasises the importance of these aspects with regard to end-of-life decisions. A study from the Netherlands [11], for example, reported that 37% of the general population approved of EUT requested by an older person who lives alone and suffers from incontinence, mobility limitations and deteriorating sensory functioning. In comparison, 85% approved in case of severe pain. A study from the United States [9] reported that while two thirds of the general population supported both AS and EUT in cases of unremitting pain, 48% (AS) and 49% (EUT) also approved in case of severe long-term care dependency. Furthermore, 36% of the respondents also approved of both AS and EUT in case of (perceiving oneself as) being a burden on the family. More recently, two studies among older adults (50+) in Austria reported that 29% would not want to live in case of severe care-dependency [14], and 42% respectively 34% approved of the availability of AS respectively EUT in such a case, if requested [15].

Additional evidence on the importance of care-dependency as a concern or motif for end-of-life decisions stems from studies analysing actual practices of AS and EUT retrospectively. For example, a study from Switzerland [16] reported that need for long-term care was the second most often reported reason for using AS (39%) after pain (58%) among those who legally died through AS. In Belgium, not wanting to be a burden on family or others was reported for 32% of all EUT requests between 2002 and 2009 [17]. Similarly, among 1917 cases of performed EUT (2002-2007), ‘dependency’ was reported in 26% as the constituting nature of psychological suffering [18]. Analogous results were recently reported for Germany, where fear of care dependency was reported in 24% (of 118) cases of AS between 2010 and 2013 [19]. Furthermore, a number of issues often closely associated with long-term care need or care-dependency were reported as central end of life concerns in more than a thousand cases of physician-AS under the Oregon Death with dignity act [20]: loosing autonomy (91%), loss of dignity (77%), losing control over bodily functions (47%), being a burden on family, friends/caregivers (42%).

Despite the empirical evidence on the relevance of (fear of) care dependency with regard to (attitudes towards) end-of-life decisions, studies assessing attitudes towards AS and EUT *among* care-dependent older adults are largely absent. This leads to the paradox situation, that the question whether or not care-dependency constitutes a palpable motif for approval of AS and EUT is answered by individuals from the general population (e.g. [11, 15]) which, most likely, are not care-dependent themselves. Thus, results from existing studies may primarily reflect negative stereotypes and fears about living as a care-dependent older adult [15]. We are aware of only one study from the early 1990s, where functionally-impaired older adults in the Netherlands were surveyed with regard their fears of the final stages of life and their opinion on hastening death [21]. In total, 34% of the older adults with functional limitations agreed (in 1994) that AS should be available for older adults if they so desire. Furthermore, interpersonal fears such as being dependent on others or being a burden to others were found to be more prominent than personal fears such as suffering or no longer recognising people in this study.

In sum, care-dependency seems to constitute an important issue with regard to the approval and usage of end-of-life decisions, yet it is clearly understudied among those most affected: care-dependent older adults.

### Determinants of attitudes towards end-of-life decisions

In many empirical studies on attitudes towards end-of-life decisions, the focus seems to lie more on the outcome itself, that is, on approval rates for varied hypothetic scenarios involving end-of-life decisions (e.g. [10, 11, 13, 22]) among the public, physicians or patients and their relatives, and less on the personal characteristics influencing differences in attitudes towards end-of-life decisions. We are not aware of any comprehensive formal theoretical framework explaining why people hold specific attitudes with regard to end-of-life decisions. Empirical studies which focus on the determinants of attitudes towards AS and EUT seem to mostly follow an ad-hoc approach, assessing a small number of predictor variables, often only socio-demographics and religiosity, whereas theoretical frameworks and theory-based generation of hypotheses remain a (laudable) exception in the field (e.g. [23, 24]). In consequence, robust empirical evidence on determinants of attitudes towards AS and EUT is available mostly for socio-demographic characteristics and religiosity. Here, studies have consistently found higher education [11, 13, 15, 22–31] and socio-cultural liberalism [13, 23, 24, 30, 32] to increase the chance of approval of AS and EUT, and religiosity [11–13, 21–33] to conversely decrease acceptance. Other socio-demographic variables such as sex, age, marital status and household size have shown less consistent or no associations with attitudes towards end-of-life decisions. Also, in studies of the general population, physical health seems not to relate directly to whether AS and EUT are approved or rejected (e.g. [11, 15, 22, 31]). Apart from these, few additional factors, if any, have been repeatedly assessed as potential determinants for attitudes towards AS and EUT. Scattered evidence nevertheless implies, for example, that depressive symptoms increase the chance of approval of AS/EUT in the general population [22], among impaired older adults [21] and among severely ill patients [9]. Indirect evidence stems from studies focussing on the characteristics of patients who died through EUT or AS, which also highlight the constituting role of ‘psychological suffering’ [18] or ‘psycho-existential’ and ‘social reasons’ [16] for utilising AS or EUT. Among the general population, concerns with regard to a future low quality of life in the last years of life have recently also been suggested to contribute to a more liberal current stance towards end-of-life decisions [15]. Mixed results have been found with regard to trust. Whereas two studies [23, 24] based on the World Value Survey have found interpersonal trust on both the individual- and the country-level associated with higher acceptance (rates) of EUT, a recent national study [15] reported the reverse result, that is, less trusting individuals were more likely to approve of the availability of AS and EUT. Interestingly, negative results have also been found with regard to trust in physicians [31] and trust in the health care system [24].

In sum, previous research has identified a number of robust correlations, notably with regard to education and religiosity, but at the same time, it is often limited to these and a few other easy to operationalise socio-demographic variables. It is unclear, however, whether these well-known associations hold when additional psychosocial factors are considered. More generally, there is a notable lack of theoretical reasoning in empirical studies as to why individuals hold specific attitudes towards end-of-life decisions.

### The current study

Based on the outlined gaps in the existing literature, this paper seeks to (1) assess attitudes towards AS and EUT among care-dependent older adults in Austria and (2) to analyse why care-dependent older adults approve or reject AS and EUT. With regard to the second goal, this study tests the impact of predictor variables grouped in five categories: (1) socio-demographics, (2) religiosity, (3) physical illness, (4) psychological distress, and (5) social isolation.

First, among *socio-demographic characteristics*, we expect strong associations with attitudes towards AS and EUT particularly with the level of education [11, 13, 15, 22–31]. This is expected due to the fact that higher educated individuals tend to hold more tolerant and liberal attitudes and value personal freedom, autonomy, and individualism more [34–36] than those who hold lower levels of education. The core value of autonomy has also been discussed as a motor of the right-to-die argument in discussions on euthanasia specifically and struggles for patient’s rights more generally [37, 38].

Second, we expect to find a consistently negative impact of *religiosity* on attitudes towards AS and EUT [11–13, 21–33]. The stronger rejection of end-of-life decisions among religious individuals is usually attributed to religious teachings – such as the Judaeo-Christian doctrine that human life is created in the image of god ([39], p40) from which an ‘intrinsic dignity’ follows, which in turn, forbids intentional killing of human beings (sanctity of life argument) – and the rejection of euthanasia and the condemnation of suicide by most religious authorities (e.g. [40]). More generally, the stronger adherence to absolute morals and socio-culturally conservative values [41] and the integration into religious social networks, reinforcing these beliefs and values [42] are also believed to contribute to a lower rate of approval of AS and EUT among religious individuals.

Third, among care-dependent older adults, we expect those who suffer more from *physical illness* to also take a more positive stance towards end-of-life decisions, particularly with regard to AS. The discourse on end-of-life decisions revolves around grave and terminal physical illnesses, such as late-stage cancer and associated uncontrollable pain (e.g. [43]), although physical decline and associated loss of control and autonomy as well as dependence on others for basic self-care have also been reported as central motivations for AS or EUT [9, 11, 18–20]. Indeed, severe physical illness may lead care-dependent older adults to experience a lack in dignity [44], that is, when they find themselves in ‘inappropriate circumstances […], incompetent, inadequate or unusually vulnerable’ [45]. In such situations, the institutionalised availability of AS could be perceived as a last resort to retain some control over one’s status. We thus expect older adults with severe physical illness to hold – on average and ceteris paribus – more favourable views on the availability and inclination to personally use AS. In contrast to AS, we expect a less clear association with EUT, as particularly care-dependent older adults with severe physical illness might at the same time be more afraid to become victims of non- and in-voluntary acts of assisted death due to their increased vulnerability (cf. slippery slope argument [39, 46, 47]).

Fourth, care dependent older adults who *suffer psychologically*, that is, individuals who feel desolate and bitterly unhappy with their life with little prospect of improvement are expected to be more open towards end-of-life decisions compared to individuals who are less deeply afflicted. Studies on actual cases of AS and EUT demonstrate the importance of psychological suffering [16, 18], and euthanasia laws and practices also acknowledge this quality explicitly (e.g. [2, 17, 18]). Suffering from mental health problems such as depression [21, 22] and/or suicidality [48–52], which reflect severe psychological distress and suffering rather than normative processes as the end of life, are expected to be associated with a more positive attitude towards end-of-life decisions, particularly towards the availability and inclination of hypothetically using AS. Additionally care-dependent older adults who suffer from perceiving themselves as mainly a burden to others [9, 20, 53] could be more approving of the availability of AS or EUT. Also, care-dependent older adults who are terrified to experience a long, painful, indignant or otherwise gruesome process of dying [54] in the near future may also perceive AS and EUT as viable alternatives of a more self-determined and controlled way to die, which’ availability might lift some of the stress associated with the prospect of dying.

Fifth, and finally, *social isolation*, that is, community-dwelling older adults who, despite depending on care from others for tasks of daily living, live alone, and those who feel isolated from others are expected to be more accepting of both AS and EUT, both of which could be perceived as a means to have more control over one’s last weeks and days in the face of an unknown and potentially lonely death [30]. In contrast, care-dependent older adults who are distrustful of others in general and of physicians specifically might still approve of AS, for example in the form of a suicide pill [22, 27] but could be more critical towards EUT [24] due to the fear of becoming a victim of non- and in-voluntary acts of assisted death (the fear at the heart of the slippery-slope argument [39, 46, 47]) or because they do not expect others to comply with their end-of-life preferences. More fundamentally, trust in physicians has been suggested as a necessary pre-condition for EUT [55].

## Methods

### Survey design

On behalf of the authors, a cross-sectional survey among Austrian care-dependent older adults (50+) was conducted by the Institute for Empirical Research (IFES, Vienna). Identifying and sampling among community-residing care-dependent older adults in Austria is not straightforward, since researchers and survey agencies in Austria do not have access to the general population register nor to administrative records containing information about a person’s level of care dependency (Pflegestufe) and the subsequent care allowance (Pflegegeld) paid to this person. Therefore, we have had IFES use a screening question (‘May I ask you: Is there someone living in your household who is officially classified as care-dependent?’ [‘Darf ich Sie fragen: Gibt es in Ihrem Haushalt jemanden, der in einer Pflegestufe ist?’]) in a large number of face-to-face interviews (*n* = 8.420) in multiple surveys selected through a multistage random sampling procedure among the general population in order to identify care-dependent older adults aged 50 year or over. Official classification of care-dependency (due to a physical or mental disability which is expected to last at least 6 months) in Austria is assessed by authorised physicians or nurses and ranges from level 1 (<65 h care need per month) to level 7 (>180 h of care need per month and constant supervision required). Using the screening question, 588 care-dependent older adults in the community (50+) were identified, of which 501 (85.2%) gave verbal consent to participate in the study. Computer-assisted personal interviews (CAPI) were carried out by IFES between June and September 2016. Participants were informed about the topic of the study and the anonymity of all personal information provided and could end the interview at all times. Eight interviews were indeed eliminated, thus resulting in a total number of 493 completed interviews. The entrusted agency (IFES) conducting the interviews is member of the World Association of Opinion and Marketing Research Professionals (ESOMAR) and bound to its code of conduct and quality standards. The conductance of this study was approved by the Ethics Committee of the University of Graz (EK-number 26–425 ex 13/14).

### Outcome variables

In this study, we assessed the attitude towards AS and EUT with regard to both general availability and hypothetic personal will to use both types of end-of-life decisions, which have been shown to differ in previous studies [27, 33, 53]. First, respondents were asked to imagine the situation of an older adult who lives alone and has been in need of assistance with activities of daily living such as eating, going to the toilet, or getting out of bed for a prolonged period of time. The attitude towards the availability of AS and EUT was operationalised with the following question [15]: ‘In case this older, care-dependent person would not want to live on, what of the following would you support:’

- ‘This person should have their wish to die fulfilled by receiving a substance for suicide’
- ‘This person should have their wish to die fulfilled by a medical doctor administering a substance which causes his/her death’

Hypothetic personal will to use either AS or EU was operationalised with a follow-up question ‘And what would you, given that you would not want to live on, consider for yourself?’

- ‘Use a provided substance in order to commit suicide’
- ‘Have a medical doctor administer a substance which causes death’

Answer categories for all four outcome variables included only yes or no in order to encourage a definitive statement.

### Predictor variables

#### Socio-demographics

Sex (male/female), age (in years), education and area of living were included. Low educational attainment referred to primary and lower secondary education, high educational attainment referred to upper secondary or higher education. Area of living included three categories: rural/village (<5000 inhabitants in municipality), town (5001-50,000 inhabitants) and city (>50,000 inhabitants).

#### Religiosity

Self-rated religiosity was operationalised with the following item: ‘How religious would you describe yourself?’ Answer categories ranged from (1) ‘very religious’ to (4) ‘not at all religious’.

#### Physical illness

Three variables were included. First, poor self-rated health referred to the question ‘Would you say your health is …’ where answer categories ranged from ‘very good’ (1) to ‘very bad’ (5). ‘Bad’ and ‘very bad’ health were classified as poor health status. Functional limitations referred to the extent of problems in the following activities of daily living (ADL): eating/drinking, get out of and into the bed or a chair, dress, use the toilet and to take a bath/shower. Answer categories ranged from 1 (‘no difficulties’) to 4 (‘not able to’) and a sum score across all five items, ranging from 5 to 20, was calculated. Poor sensory functioning was measured with an item from WHOQOL-OLD, a survey instrument designed to measure quality of life among older adults: ‘How would you rate your sensory functioning (e.g. hearing, vision, taste, smell, touch)?’. Answer categories ranged from ‘very poor’ (1) to ‘very good’ (5). Answer categories ‘very poor’ and ‘poor’ were classified as poor sensory functioning.

#### Psychological distress

This included four variables*.* First, fear of death was measured with the death and dying subscale from WHOQOL-OLD comprised of 4 items (α = 0.80, range = 4-20) such as ‘How much do you fear being in pain before you die?’ or ‘How much are you afraid of not being able to control your death?’ each with answer categories ranging from not at all (1) to an extreme amount (5). Second, perceived burdensomeness was measured with the subscale of the same name in the Interpersonal Needs Questionnaire short form (INQ-10) [56, 57] comprised of 5 items (α = 0.88) such as ‘These days I think my death would be a relief to the people in my life’ or ‘These days, I think I make things worse for the people in my life’. Answer categories ranged from 1 (not at all true for me) to 7 (very true for me) and thus the scale ranged from 5 (low) to 35 (high). Third, depressed affect, a subscale of the Center for Epidemiologic Studies Depression Scale (CES-D) [58] was measured with the following three items (α = 0.70) indicating how respondents felt in recent time: ‘I felt depressed’; ‘I felt lonely’; ‘I felt sad’. Answer categories ranged from rarely or none of the time (1) to most or all of the time (4), resulting in a sum index ranging from 3 (low) to 12 (high). Fourth, suicide ideation was measured with the Paykel Scale [59] resulting in three categories: no suicide ideation, passive suicide ideation (thoughts that life is not worth living), and active suicide ideation (thoughts about harming oneself).

#### Social isolation

This included four variables. First, living alone (no/yes). Second, thwarted belonging measures the lack of perceived connectedness to others and refers to a subscale of the INQ-10 including 5 items such as ‘These days, I feel disconnected from other people’ or ‘These days, I feel like I belong’ (α = 0.73). Answer categories ranged from 1 (not at all true for me) to 7 (very true for me) and thus the scale ranged from 5 (low) to 35 (high). Third, social trust was measured with five items (e.g.: ‘In general, people can be trusted’; ‘Most people try to take advantage of others’) (α = 0.71) based on a short-scale of social trust (KUSIV3 [60]). Fourth, trust in physicians was measured with four items (e.g. ‘In general, doctors can be trusted’, ‘Doctors are more interested in making money than in their patients’) (α = 0.63) [61]. Answer categories for items of both social trust and trust in physicians ranged from 1 (completely agree) to 4 (completely disagree). Thus, the scale of social trust ranged from 5 (low) to 20 (high) and the scale of trust in medical doctors ranged from 4 (low) to 16 (high).

### Analysis

Several variables showed non-negligible missing values (>1%): availability of EUT (18.5%), will to use EUT (18.1%), availability of AS (16.4%), perceived burdensomeness (15.8%), will to use AS (13.8%), trust in doctors (13.6%), thwarted belonging (10.1%), fear of dying (8.9%), social trust (7.7%), and suicide ideation (5.7%). Patterns of item non-response were analysed and showed a clear gradient with regard to area of living and health (see results section). Thus, missing values were considered to be rather missing at random (MAR), that is, their missingness is related to other characteristics such as a poor physical health and thus potentially observable variables and not primarily to the values of the variable in question [62]. Since list-wise deletion would in this case lead to both biased estimates and lower statistical power, we instead applied a multiple imputation procedure using chained equations in R-package *mice* (v2.25) resulting in 50 imputed datasets. In order to approximate MAR, we utilised all predictor variables in the imputation model [63].

Bivariate analyses of associations between predictor and outcome variables were based on Chi^2^-tests for categorical and linear regression models for continuous predictors. For multivariate analyses of the dichotomous outcome variables, logistic regression analysis was used, the results of which were pooled across the imputed datasets according to Rubin’s rule [64] in order to correctly account for uncertainty associated with the multiply imputed values. Continuous predictor variables were mean-centred and divided by 2 standard deviations in order to make them more easily comparable with effect sizes from binary categorical variables [65]. All data analysis was performed using R: A language and environment for statistical computing (3.3.2) [66].

## Results

### Sample characteristics

58.2% of the sample were women and average age was 73.2 years (SD = 11.0, range = 50-98). In total, 28.6% of the respondents lived alone, with women being more likely to live alone (36.2%) than men (18.0%) (χ^2^ = 19.6, df = 1, *p* < 0.001). 22.9% lived in rural areas or villages, 18.7% in towns and 58.4% lived in cities. Details of sample characteristics for all variables can be found in Table 1.

**Table 1:** Sample characteristics

Variable	n (%)	n (mean, SD)
Sex		
Men	206 (41.8)	
Women	287 (58.2)	
Age (years)		493 (74.2, 11.0)
Education		
Low	405 (82.2)	
High	88 (17.9)	
Area of living		
Rural/village	113 (22.9)	
Town	92 (18.7)	
City	288 (58.4)	
Religiosity		
Very religious	165 (33.8)	
Rather religious	212 (43.4)	
Rather not religious	73 (15.0)	
Not at all religious	38 (7.7)	
Poor self-rated health		
No	379 (77.4)	
Yes	111 (22.7)	
Functional limitations (ADL)		491 (9.2, 3.0)
Poor sensory functioning		
No	389 (78.9)	
Yes	104 (21.1)	
Fear of dying		449 (8.8, 3.1)
Perceived burdensomeness		415 (13.2, 7.3)
Depressed affect		489 (5.6, 1.8)
Suicide ideation		
None	275 (59.2)	
Passive	143 (30.8)	
Active	47 (10.1)	
Living alone		
No	352 (71.4)	
Yes	141 (28.6)	
Thwarted belonging		443 (16.6, 6.9)
Social trust		455 (14.0, 2.8)
Trust in doctors		426 (11.3, 2.4)

Unweighted data, SD standard deviation

### Item non-response

We found item non-response consistently and substantially associated with the size of the community of residence, i.e. respondents in rural areas of Austria were significantly less likely to provide valid answers. With regard to the availability of AS and EUT, for example, 31.9% and 32.7% of the rural inhabitants provided no valid answer, compared to only 8.7% and 10.8% of those living in cities (AS: χ^2^ = 34.1, df = 2, *p* < 0.001; EUT: χ^2^ = 29.3, df = 2, p < 0.001). Similar patterns of missingness associated with residence were also found for will to use AS, will to use EUT, perceived burdensomeness, thwarted belonging, trust in physicians, social trust, fear of death and suicide ideation. Additionally, missing values among the independent variables were more often found among older adults (e.g. perceived burdensomeness: F = 7.9, df = 492, *p* = 0.005), those with poor subjective health (e.g. social trust: χ^2^ = 6.7, df = 1, *p* = 0.010) and those with more ADL restrictions (e.g. suicide ideation: F = 6.9, df = 489, *p* = 0.009).

### Descriptive analysis

Rates of approval in the four dependent variables were highly similar. 26.0% and 24.8% of the respondents approved of the availability of AS respectively indicated the will to hypothetically use AS. 57.6% and 61.5% rejected the availability of AS respectively negated the will to use AS, and 16.4% and 13.8% provided no clear yes or no answer. With regard to EUT, 25.0% approved of its availability and 24.8% indicated the will to hypothetically use it. 56.6% and 57.2% rejected EUT and 18.5% and 18.1% did not a valid yes or no answer. Tetrachoric correlation coefficients – which assume a latent trait of approval of end-of-life decisions – between the outcome variables were positive and substantial: availability AS and will to use AS = 0.80; availability EUT and will to use EUT = 0.75. This means that care-dependent older adults who approved of the availability of either AS or EUT were also highly likely to report the personal will to use them for themselves. Approval of availability and will to use across AS and EUT were also closely associated (*r* = 0.46-0.66).

Additional file 1: Table S1 and S2 show results from bivariate statistical analysis. With regard to both availability and hypothetical use of AS, we found strong and statistically significant (*p* < 0.05) bivariate associations in particular for area of living, religiosity, self-rated health, fear of death, suicide ideation and trust in physicians. For example, 46.6% and 39.7% of the sampled care-dependent older adults who rated their own health as poor approved of the availability of AS respectively indicated to hypothetically use AS compared to only 26.6% respectively 25.7% among those with better self-rated health. For EUT, we similarly found strong bivariate associations with area of living, religiosity, fear of death, suicide ideation and trust, but also with regard to perceived burdensomeness and living alone. For example, among very religious individuals, only 19.3% approved of the availability of EUT compared to 50.0% of those who described themselves as not at all religious. Similar sized differences showed for example for those who lived alone, where 43.7% indicated to hypothetically use EUT compared to 24.6% among those who lived with others.

### Multivariate analysis

Table 2 shows the results of the fully adjusted models for each of the four outcomes. Among socio-demographic variables, no strong associations showed expect for area of living. Noteworthy, this includes education. Respondents from more urban areas had 2.5 times the odds to approve of AS; with regard to EUT, effects were less consistent in comparison. In contrast to the bivariate analyses, the effect of religiosity under full model adjustment was statistically significant only with regard to the availability of EUT – non-religious individuals had a two to four time higher chance to approve – but not with regard to utilisation of EUT or AS. With regard to physical illness, care-dependent older adults who rated their own health as poor were twice as likely to approve of AS. Apart from this, no strong associations showed between measures of physical illness and attitudes towards end-of-life decisions. Concerning psychological distress, a result consistent across all four outcomes was that respondents with a profound fear of death had a 2.0-2.5 times increased chance to approve of both the availability and personal utilisation of AS and EUT compared to those with low levels of fear of death. No clear associations showed for depressed affect and perceived burdensomeness in the fully adjusted model. Furthermore, active suicide ideation was clearly associated with attitudes towards AS. Respondents who reported to have actively thought about committing suicide in the last 6 months had a 2.5 higher chance to approve of the availability of AS, and a 3.1 higher chance to report the will to personally use AS. Finally, respondents who lived alone had a 2.5 times increased chance to approve of the availability of EUT and a 4 times higher chance to indicate the will to personally use EUT. Those who felt strongly isolated from others were somewhat more likely to disapprove of the availability of AS. A negative association showed between trust in doctors and attitudes towards AS. Overall, pseudo-R^2^ (Nagelkerke) values indicated good and highly similar model fit for the full models between 0.18-0.20.

**Table 2:** Results from logistic regression analysis for attitudes towards assisted suicide and euthanasia

	Availability of assisted suicide (yes = 1)	Hypothetic utilisation of assisted suicide (yes = 1)	Availability of euthanasia (yes = 1)	Hypothetic utilisation of euthanasia (yes = 1)
	OR (CI-95)	OR (CI-95)	OR (CI-95)	OR (CI-95)
Socio-demographics				
Women (Ref. = Men)	0.70 (0.43-1.13)	0.75 (0.46-1.22)	1.07 (0.64-1.78)	0.83 (0.51-1.35)
Age (2SDs = 22.0)	1.08 (0.65-1.79)	*0.64 (0.39-1.06)*	0.73 (0.44-1.21)	0.81 (0.49-1.35)
High education (Ref. = low)	1.54 (0.83-2.85)	1.33 (0.73-2.42)	1.07 (0.58-1.97)	1.18 (0.65-2.16)
Town/small city (Ref. = rural/village)	*2.13 (0.97-4.65)*	***2.53 (1.09-5.87)***	1.55 (0.67-3.58)	1.20 (0.54-2.64)
Large city (Ref. = rural/village)	***2.22 (1.12-4.40)***	***2.24 (1.05-4.78)***	*1.91 (0.96-3.78)*	1.67 (0.84-3.32)
Religiosity				
Rather religious (Ref. = very religious)	1.35 (0.77-2.35)	*1.73 (0.97-3.08)*	***2.24 (1.26-3.97)***	0.82 (0.46-1.44)
Rather not religious (Ref. = very religious)	1.31 (0.63-2.72)	*1.94 (0.92-4.08)*	*1.99 (0.92-4.31)*	1.56 (0.76-3.22)
Not at all religious (Ref. = very religious)	1.36 (0.53-3.49)	1.61 (0.64-4.09)	***4.16 (1.66-10.4)***	1.68 (0.70-2.60)
Physical illness				
Poor self-rated health (Ref. = no)	***1.91 (1.05-3.45)***	*1.73 (0.94-3.16)*	0.73 (0.39-1.38)	1.41 (0.76-2.60)
Functional limitations (2SDs = 5.9)	0.95 (0.54-1.67)	0.96 (0.53-1.72)	0.78 (0.44-1.38)	0.82 (0.45-1.48)
Poor sensory functioning (Ref. = no)	1.08 (0.60-1.96)	0.92 (0.52-1.65)	0.72 (0.39-1.32)	*1.01 (0.57-1.80)*
Psychological distress				
Fear of death (2SDs = 6.2)	***2.58 (1.50-4.42)***	***2.16 (1.21-3.87)***	***2.11 (1.20-3.69)***	***2.56 (1.45-4.53)***
Perceived burdensomeness (2SDs = 14.5)	1.45 (0.68-3.06)	1.32 (0.64-2.73)	1.37 (0.62-3.02)	1.41 (0.66-3.02)
Depressed affect (2SDs = 3.7)	0.91 (0.52-1.60)	0.64 (0.37-1.13)	1.59 (0.89-2.81)	0.90 (0.52-1.58)
Passive suicide ideation (Ref. = none)	0.92 (0.49-1.74)	1.04 (0.56-1.91)	0.79 (0.43-1.48)	0.90 (0.47-1.73)
Active suicide ideation (Ref. = none)	***2.52 (1.08-5.89)***	***3.10 (1.37-7.04)***	1.59 (0.70-3.59)	*2.12 (0.91-4.91)*
Social isolation				
Living alone (Ref. = living with others)	*1.71 (0.93-3.12)*	1.47 (0.82-2.65)	***2.54 (1.39-4.63)***	***3.99 (2.19-7.26)***
Thwarted belonging (2SDs = 13.8)	*0.50 (0.22-1.13)*	0.64 (0.29-1.41)	0.96 (0.42-2.21)	0.69 (0.31-1.53)
Social trust (2SD = 5.7)	1.30 (0.78-2.19)	1.50 (0.87-2.56)	0.75 (0.45-1.25)	1.20 (0.71-2.03)
Trust in doctors (2SDs = 4.8)	*0.58 (0.33-1.03)*	***0.55 (0.32-0.97)***	0.85 (0.50-1.44)	0.68 (0.39-1.18)
AIC/Pseudo-R^2^ (Nagelkerke)	582/0.186	573/0.177	579/0.197	573/0.197

*N* = 493, unweighted data, *OR* odds ratio, CI-95 = 95% confidence interval, 2SDs = 2 standard deviations, AIC = Akaike Information Criterion. Bold font indicates *p* < 0.05, italic font *p* < 0.1. Continuous variables are mean centred and divided by two standard deviations

## Discussion

Long-term care dependency in old age is a relevant issue for attitudes towards AS and EUT [9, 11, 15], but it has received limited attention so far. Particularly, there are two shortcomings we attempted to address with this article. First, to our knowledge, there is hardly any empirical evidence with regard to the rate of approval of end-of-life decisions among care-dependent older adults available. Second, research focussing on the determinants of attitudes towards AS and EUT is often limited to socio-demographic factors and religiosity.

In our study, we found a quarter of the surveyed care-dependent older adults (50+) to both approve of the availability (in case of care-dependency) of and to report will to hypothetically use AS and EUT. This is an expectedly lower rate of approval compared to other studies surveying the general population from the Netherlands [11, 21] and the U.S. [9], but also in comparison to a recent study from Austria [15] which used the identical items to measure the approval of the availability of AS and EUT in case of care-dependency among the general older population (50+). The latter study found 42% respectively 34% approval of AS respectively EUT in case of long-term care dependency among the general older population in Austria. There are several potential explanations for the latter gap. First, the gap could be due to differences in the living situation. Care-dependent older adults were on average older (mean = 73.2 years) than respondents from the general older population (mean = 65.3 years) from the previous study and, obviously, in poorer health. Thus, care-dependent older adults in comparison to the general older population might have had more chance and incentives to consider and critically evaluate potential end-of-life decisions. A negative effect of age on approval of AS and EUT has been reported in some previous studies (e.g. [23, 28, 33]), whereas none or positive findings with regard to age, that is, increased approval in older age, showed in others (e.g. [11, 15, 21, 22, 26, 30]). Second, the difference might also reflect greater fears among more vulnerable care-dependent older adults with regard to being devaluated or pressured into considering ending their lives. A third reason for the gap in approval between the two samples of general and care-dependent older adults could be also that the respondents from the latter were more religious. In the sample of care-dependent older adults, 33.5% reported to be ‘very religious’ compared to only 17.3% among the general older adults. However, controlling the impact of both age and religiosity statistically in calculations (not shown) using data from both the sample of older adults [15] and the sample of care-dependent older adults of the current study indicated that only a small part of the difference in the approval rate of AS and EUT between the studies could be attributed to the higher level of religiosity among the sampled care-dependent older adults and that the difference in average age did not matter. This leaves another explanation for the gap in approval between the two Austrian samples, that is, it could primarily reflect fears and negative stereotypes among the general population about the alleged low quality of life as a care-dependent older adult [15].

The consistency with regard to the rate of approval among care-dependent older adults between both the type of end-of-life decision (AS versus EUT) and between availability and personal use found in the current study is also noteworthy. Previous studies have reported both differences [13, 15, 22, 31] and similarities [9] with regard to the approval rates of AS and EUT and differences between general availability and personal will to use [27, 33, 53] end-of-life practices. The high correlation with regard to approval between availability and personal usage could be interpreted insofar as – unlike younger and healthier individuals for whom end-of-life decisions refer to a rather distant, value-laden issue than actual practices – care-dependent older adults are less likely to differentiate between the issue of the general availability and personal usage of AS and EUT because of their living situation close(r) to the end of life.

With regard to the determinants of attitudes towards AS and EUT, the limited role of religiosity in the fully adjusted models for all outcome variables except availability of EUT is noteworthy, particularly against the backdrop of the ascribed importance of religiosity in virtually all studies on the topic [11–13, 15, 21–33]. Indeed, we also found considerable bivariate associations between self-reported religiosity and all four outcome variables, yet these diminished under full model adjustment. Thus, the in comparison to most of the previous studies larger number of potential determinants assessed in this paper may partially account for the diminished effect of religiosity. In particular, religious persons in our study were also somewhat more trustful, less likely to live alone and reported lower levels of fear of death than those who described themselves as ‘rather not religious’. The in comparison lower impact of religiosity could also be due to the sample itself. Care-dependent older adults in their last years of life may, in comparison to the general population, have a more pragmatic dealing with questions regarding the end-of-life than younger persons, for which these issues might be more distant or mainly of ideological nature. A similar interpretation may apply with regard to education, for which we, again different to many studies [11, 22–31] did not find a clear association with attitudes towards AS or EUT. The rural-urban gap we found with regard to AS, that is, care-dependent respondents in urban areas where individualistic orientations tend to be more pronounced [67] were also considerably more likely to endorse AS than their counterparts living in more conservative-minded rural areas, is not surprising, although rarely documented [28].

With regard to role of physical illness, we found mixed empirical support of our expectation that care-dependent older adult with poor physical health are more likely to approve of AS than those in better health. Although we found some modest effects with regard to self-rated health, no associations showed with regard to the degree of functional limitations concerning daily activities of living or sensory deprivation. In the light of the results of previous studies, which assessed the general (older) population and had generally found no strong associations between physical health status and attitudes towards end-of-life decisions [11, 13, 15, 22, 31], the fact that we found only the self-rated health item associated only with AS points to an alternative interpretation. The item used to measure self-rated health has been credited to measure physical health status up to old age [68], but subjective well-being, life satisfaction and positive affect are also thought to be incorporated [69] and even gain importance for ‘self-rated health’ with advancing age [70]. Thus, we consider the effect to be more likely a methodological issue respectively reflecting psychological states rather than the role of physical health proper.

With regard to psychological distress, strong evidence was found for the impact of active suicide ideation and fear of dying, which both have rarely been explicitly tested as potential determinants of attitudes towards end-of-life decisions [53, 71]. The impact of active suicide ideation, that is having considered to end one’s life in the last 6 months, a distinct marker of psychological distress [50], highlights the role of severe psychological suffering for attitudes towards AS, and underlines the troubling issue whether clinically depressed or suicidal individuals should be able to participate in institutionalised assisted suicide if it was legal [72, 73].

More than active suicide ideation, fear of dying, that is worrying about the way one will die, particularly with regard to suffering from pain [71] and having little control over the process of dying, was consistently associated with approval of both availability of and personal inclination to us both AS and EUT. Thus, fears related to the process of dying and the wish to control this process, i.e. to cope with the threat of a potential dreadful end-of-life in the (near) future represents a central motivation for a more positive stance in the present day toward end-of-life decisions such as AS and EUT. Arguably, this is because both AS and EUT can be perceived as providing a more self-determined and controlled way to die, irrespectively of the fact that both AS and EUT are illegal acts in Austria. More generally, medical and social care providers as well as informal caregivers should thus strife to provide counselling, comfort and compassionate end-of-life care in order to reduce fears of the process of dying, as far as this is possible.

With regard to social isolation, we found that the rather few care-dependent older adults who lived alone were far more in favour of EUT – particularly with regard to the inclination to use it for themselves rather than with regard to its more general availability – but less so with regard to AS than respondents who lived with others. This could be driven by a fear of those who live alone to also die alone [30] and may reflect a preference to rather rely on a medical professional to oversee one’s last hours. Finally and against our expectations, lack of trust towards others in general and towards physicians specifically was not associated with EUT. Thus, preference for or against euthanasia and personal inclination to use it (hypothetically) seems not to require high levels of trust in physicians in general, at least among care-dependent older adults who likely have regular contact with physicians due to their precarious health state. It could be that for the intimate and in Austria illegal act of EUT, the level of trust towards their own general practitioner is crucial and not the level of trust towards physicians more generally. In contrast, respondents with low trust in medical doctors were, interestingly, at the same time somewhat more likely to approve of the availability and to report personal will to use AS. Similar negative effects of trust in doctors [24, 31] have been reported previously and might be interpreted as the readiness of care-dependent older adults to take end-of-life decisions literarily in to their own hands – AS rather than EUT where doctors were explicitly mentioned in the item – when doctors (or the medical system more generally) are perceived as not trustworthy to comply with their wishes.

### Strengths and limitations

The strengths of this study are the nation-wide sample of care-dependent older adults, the use of validated measurements, four outcome variables covering both availability and intent to use both AS and EUT, and the number of tested potential determinants of attitudes towards AS and EUT. But there are also several noteworthy limitations to this study. First, care-dependent older adults living in cities (>50,000 inhabitants) were oversampled and those with the highest levels of care need likely under-sampled, which may bias our results. Indeed, surveying the oldest old as well as care-dependent older adults is often associated with survey design issues [74]. Unfortunately, potentially compensating post-stratification weights could not be computed since information on the number of care-dependent older adults living in the community and their characteristics (age, sex, care-level, residence etc.) is not available in Austria. Second, we analysed cross-sectional survey data from which no strong causal conclusions can be drawn. However, given that we analysed attitudes towards AS and EUT as outcome, potential reversed causality seems an issue of limited concern. Third, potentially important predictors which were not included in the survey could have affected our results. This includes for example experience of severe pain, the importance of personal freedom and autonomy or trust towards the state’s administration. Third, and finally, missing values in both the outcome and predictor variables were substantial – similar rates of non-response have been reported before with regard to questions involving end-of-life decisions in Austria [13, 15] though – and induce a certain degree of uncertainty into our analyses for a number of variables. We sought to address the issue by analysing patterns of missingness, which highlighted the role of area of living (rural-urban gap) and physical illness, and a subsequent multiple imputation procedure utilising all predictor variables in order to approximate the constituting assumption of MAR. If, however, missing values were in contrast mostly related to the respective value itself, this would also bias our results to an unknowable degree.

## Conclusion

In conclusion, we found that among care-dependent older adults in Austria, a substantial minority of about one quarter approves of AS and EUT. Attitudes toward AS were more favourable among care-dependent older adults living in urban areas, those who did not trust physicians, those who reported active suicide ideation, and individuals with a strong fear of dying. With regard to EUT, living alone, religiosity and fear of dying were the central determinants of acceptance.

## Additional file

Additional file 1: This manuscript is linked to an additional file (additional_file1), a MS-Word data-file containing two tables (Additional Table S1 and S2) showing the results of bivariate associations between predictor and outcome variables. (DOCX 20 kb)

## Abbreviations

ADL: Activities of daily living
AS: Assisted suicide
CAPI: Computer-assisted personal interviews
df: Degrees of freedom
EUT: Euthanasia
IFES: Institute for empirical research (Vienna)
INQ-10: Interpersonal needs questionnaire short form
MAR: Missing at random
n: Sample size
p: *P*-value
r: Pearson’s correlation coefficient
SD: Standard deviation
StGB: Strafgesetzbuch [Austrian Criminal Code]
χ^2^: Chi^2^-test
