# Supplementary material for: Attitudes towards assisted suicide and euthanasia among care-dependent older adults (50+) in Austria: the role of socio-demographics, religiosity, physical illness, psychological distress, and social isolation
Source: BMC Med Ethics. 2017 Dec 7;18:71. doi: 10.1186/s12910-017-0233-6 (PMC5719645; doi:10.1186/s12910-017-0233-6)
Supplement: Additional file 1: — This manuscript is linked to an additional file (additional_file1), a MS-Word data-file containing two tables (Additional Table S1 and S2) showing the results of bivariate associations between predictor and outcome variables. (DOCX 20 kb) [file 12910_2017_233_MOESM1_ESM.docx]

Additional table 1

|  | Availability of assisted suicide | |  |  | Hypothetic utilisation of assisted suicide | |  |  |
| --- | --- | --- | --- | --- | --- | --- | --- | --- |
|  | No | Yes |  |  | No | Yes |  |  |
|  | n (%) or n (mean) | n (%) or n (mean) | χ², df, p-value | F, df, p-value | n (%) or n (mean) | n (%) or n (mean) | χ², df, p-value | F, df, p-value |
| SOCIO-DEMOGRAPHICS |  |  |  |  |  |  |  |  |
| Men | 112 (64.7) | 61 (35.3) | 2.5, 1, 0.118 |  | 118 (67.4) | 57 (32.6) | 2.2, 1, 0.141 |  |
| Women | 172 (72.0) | 67 (28.0) |  |  | 185 (74.0) | 65 (26.0) |  |  |
| Age | 284 (73.5) | 128 (73.9) |  | 0.2, 410, 0.700 | 303 (74.8) | 122 (71.9) |  | 6.3, 423, 0.012 |
| Low education | 236 (70.4) | 99 (29.6) | 1.9, 1, 0.166 |  | 252 (72.2) | 97 (27.8) | 0.8, 1, 0.373 |  |
| High education | 48 (62.3) | 29 (37.7) |  |  | 51 (67.1) | 25 (32.9) |  |  |
| Rural/village | 60 (77.9) | 17 (22.1) | 3.6, 2, 0.162 |  | 72 (82.8) | 15 (17.2) | 7.1, 2, 0.030 |  |
| Town/small city | 49 (68.1) | 23 (31.9) |  |  | 51 (68.9) | 23 (31.1) |  |  |
| Large city | 175 (66.5) | 88 (33.5) |  |  | 180 (68.2) | 84 (31.8) |  |  |
| RELIGIOSITY |  |  |  |  |  |  |  |  |
| Very religious | 114 (78.1) | 32 (21.9) | 10.6, 3, 0.014 |  | 120 (81.1) | 28 (18.9) | 11.8, 3, 0.008 |  |
| Rather religious | 116 (65.5) | 61 (34.5) |  |  | 121 (68.0) | 57 (32.0) |  |  |
| Rather not religious | 31 (57.4) | 23 (42.6) |  |  | 39 (61.9) | 24 (38.1) |  |  |
| Not at all religious | 20 (62.5) | 12 (37.5) |  |  | 22 (62.9) | 13 (37.1) |  |  |
| PHYSICAL ILLNESS |  |  |  |  |  |  |  |  |
| Non-poor self-rated health | 234 (73.4) | 85 (26.6) | 12.9, 1, <0.001 |  | 243 (74.3) | 84 (25.7) | 6.3, 1, 0.008 |  |
| Poor self-rated health | 50 (53.8) | 43 (46.2) |  |  | 58 (60.4) | 38 (39.6) |  |  |
| Functional limitations | 284 (9.0) | 127 (9.6) |  | 3.1, 409, 0.080 | 302 (9.1) | 122 (9.4) |  | 0.9, 422, 0.344 |
| Non-poor sensory functioning | 232 (71.4) | 93 (28.6) | 4.3, 1, 0.038 |  | 242 (72.7) | 91 (27.3) | 1.4, 1, 0.232 |  |
| Poor sensory functioning | 52 (59.8) | 35 (40.2) |  |  | 61 (66.3) | 31 (33.7) |  |  |
| PSYCHOLOGICAL DISTRESS |  |  |  |  |  |  |  |  |
| Fear of death | 261 (8.3) | 122 (10.0) |  | 23.7, 381, <0.001 | 279 (8.5) | 115 (9.6) |  | 10.7, 392, 0.001 |
| Perceived burdensomeness | 253 (13.0) | 112 (14.6) |  | 3.6, 363, 0.060 | 260 (13.0) | 110 (14.3) |  | 2.5, 368, 0.116 |
| Depressed affect | 283 (5.4) | 128 (5.9) |  | 6.1, 409, 0.014 | 302 (5.6) | 122 (5.7) |  | 0.5, 422, 0.466 |
| No suicide ideation | 167 (73.9) | 59 (26.1) | 10.0, 12, 0.007 |  | 176 (75.2) | 58 (24.8) | 11.7, 2, 0.003 |  |
| Passive suicide ideation | 89 (69.0) | 40 (31.0) |  |  | 98 (72.6) | 37 (27.4) |  |  |
| Active suicide ideation | 22 (50.0 | 22 (50.0) |  |  | 22 (50.0) | 22 (50.0) |  |  |
| SOCIAL ISOLATION |  |  |  |  |  |  |  |  |
| Living with others | 211 (70.3) | 89 (29.7) | 1.0, 1, 0.314 |  | 213 (70.8) | 88 (29.2) | 0.1, 1, 0.71 |  |
| Living alone | 73 (65.2) | 39 (34.8) |  |  | 90 (72.6) | 34 (27.4) |  |  |
| Thwarted belonging | 264 (16.8) | 123 (17.0) |  | 0.1, 385, 0.812 | 280 (16.5) | 113 (17.3) |  | 1.0, 391, 0.327 |
| Social trust | 268 (14.1) | 124 (13.8) |  | 0.8, 390, 0.360 | 283 (13.9) | 120 (13.9) |  | 0.01, 401, 0.784 |
| Trust in doctors | 251 (11.5) | 120 (10.8) |  | 7.7, 369, 0.006 | 265 (11.5) | 115 (10.8) |  | 7.3, 378, 0.007 |

Unweighted data, Χ² = Chi²-value, F = F-value from bivariate regression analysis, df = degrees of freedom. For categorical predictor variables, Χ²-tests were performed; for continuous predictor variables bivariate linear regression models were estimated.

Additional table 2

|  | Availability of euthanasia | |  |  | Hypothetic utilisation of euthanasia | |  |  |
| --- | --- | --- | --- | --- | --- | --- | --- | --- |
|  | No | Yes |  |  | No | Yes |  |  |
|  | n (%) or n (mean) | n (%) or n (mean) | χ², df, p-value | F, df, p-value | n (%) or n (mean) | n (%) or n (mean) | χ², df, p-value | F, df, p-value |
| SOCIO-DEMOGRAPHICS |  |  |  |  |  |  |  |  |
| Men | 115 (70.1) | 49 (29.9) | 0.1, 1, 0.795 |  | 115 (69.3) | 51 (30.7) | 0.1, 1, 0.848 |  |
| Women | 164 (68.9) | 74 (31.1) |  |  | 167 (70.2) | 71 (29.8) |  |  |
| Age | 279 (74.3) | 123 (73.2) |  | 0.8, 400, 0.379 | 282 (74.1) | 122 (73.5) |  | 0.3, 402, 0.600 |
| Low education | 226 (69.1) | 101 (30.9) | 0.1, 1, 0.792 |  | 232 (70.3) | 98 (29.7) | 0.2, 1, 0.643 |  |
| High education | 53 (70.7) | 22 (29.3) |  |  | 50 (67.6) | 24 (32.4) |  |  |
| Rural/village | 60 (78.9) | 16 (21.1) | 4.6, 2, 0.010 |  | 57 (73.1) | 21 (26.9) | 1.1, 2, 0.573 |  |
| Town/small city | 49 (71.0) | 20 (29.0) |  |  | 51 (72.9) | 19 (27.1) |  |  |
| Large city | 170 (66.1) | 87 (33.9) |  |  | 174 (68.0) | 82 (32.0) |  |  |
| RELIGIOSITY |  |  |  |  |  |  |  |  |
| Very religious | 117 (80.7) | 28 (19.3) | 16.3, 3, <0.001 |  | 108 (74.5) | 37 (25.5) | 14.9, 3, 0.002 |  |
| Rather religious | 112 (65.9) | 58 (34.1) |  |  | 123 (74.5) | 42 (25.5) |  |  |
| Rather not religious | 33 (64.7) | 18 (35.3) |  |  | 34 (55.7) | 27 (44.3) |  |  |
| Not at all religious | 17 (50.0) | 17 (50.0) |  |  | 16 (50.0) | 16 (50.0) |  |  |
| PHYSICAL ILLNESS |  |  |  |  |  |  |  |  |
| Non-poor self-rated health | 217 (70.0) | 93 (30.0) | 0.4, 1, 0.546 |  | 227 (72.8) | 85 (27.2) | 6.34, 1, 0.012 |  |
| Poor self-rated health | 60 (66.7) | 30 (33.3) |  |  | 53 (58.9) | 37 (41.1) |  |  |
| Functional limitations | 279 (9.2). | 122 (9.2) |  | 0.1, 399, 0.817 | 281 (9.2) | 122 (9.4) |  | 0.5, 401, 0.497 |
| Non-poor sensory functioning | 221 (69.7) | 96 (30.3) | 0.1, 1, 0.792 |  | 225 (71.0) | 92 (29.0) | 1.0, 1, 0.326 |  |
| Poor sensory functioning | 58 (68.2) | 27 (31.8) |  |  | 57 (65.5) | 30 (34.5) |  |  |
| PSYCHOLOGICAL DISTRESS |  |  |  |  |  |  |  |  |
| Fear of death | 260 (8.5) | 112 (9.7) |  | 10.8, 370, 0.001 | 266 (8.5) | 112 (9.6) |  | 10.2, 376, 0.001 |
| Perceived burdensomeness | 248 (12.9) | 105 (15.1) |  | 7.1, 351, 0.008 | 249 (13.1) | 103 (14.6) |  | 2.98, 350, 0.085 |
| Depressed affect | 278 (5.4) | 123 (6.0) |  | 11.1, 399, 0.001 | 282 (5.5) | 122 (5.9) |  | 5.4, 402, 0.021 |
| No suicide ideation | 163 (73.1) | 60 (26.9) | 7.5, 2, 0.024 |  | 168 (73.4) | 61 (26.6) | 6.8, 2, 0.033 |  |
| Passive suicide ideation | 87 (69.0) | 39 (31.0) |  |  | 86 (69.9) | 37 (30.1) |  |  |
| Active suicide ideation | 20 (51.3) | 19 (48.7) |  |  | 23 (53.5) | 20 (46.5) |  |  |
| SOCIAL ISOLATION |  |  |  |  |  |  |  |  |
| Living with others | 208 (73.0) | 77 (27.0) | 5.9, 1, 0.015 |  | 215 (75.4) | 70 (24.6) | 14.6, 1, <0.001 |  |
| Living alone | 71 (60.7) | 46 (39.3) |  |  | 67 (56.3) | 52 (43.7) |  |  |
| Thwarted belonging | 261 (16.5) | 116 (17.3) |  | 1.2, 375, 0.268 | 265 (16.7) | 112 (17.0) |  | 0.1, 375, 0.725 |
| Social trust | 265 (14.2) | 119 (13.4) |  | 6.2, 382, 0.013 | 265 (14.0) | 116 (13.6) |  | 2.0, 379, 0.158 |
| Trust in doctors | 248 (11.5) | 112 (10.9) |  | 6.1, 358, 0.014 | 251 (11.5) | 111 (10.8) |  | 6.0, 360, 0.015 |

Unweighted data, Χ² = Chi²-value, F = F-value from bivariate regression analysis, df = degrees of freedom. For categorical predictor variables, Χ²-tests were performed; for continuous predictor variables bivariate linear regression models were estimated.
